# Impact of Diabetic Foot Multidisciplinary Unit on Incidence of Lower-Extremity Amputations by Diabetic Foot

**DOI:** 10.3390/jcm12175608

**Published:** 2023-08-28

**Authors:** Ángel Ortiz-Zúñiga, Jordi Samaniego, Betina Biagetti, Nicolás Allegue, Anna Gené, Andrea Sallent, Almudena Crespo, Jordi Serracanta, Carme Torrents, Daniela Issa, Danilo Rivas, Maria Teresa Veintemillas, Núria Fernández-Hidalgo, Rosa Busquets, Josep Royo, Cristina Hernández

**Affiliations:** 1 Endocrinology and Nutrition Department, Vall d’Hebron Hospital Campus, 08035 Barcelona, Spain; angelmichael.ortiz@vallhebron.cat (Á.O.-Z.); jordi.samaniego@vallhebron.cat (J.S.); betinaloys.biagetti@vallhebron.cat (B.B.); 2 Diabetes and Metabolism Research Unit, Vall d’Hebron Research Institute, 08035 Barcelona, Spain; 3 CIBER de Diabetes y Enfermedades Asociadas (CIBERDEM), Instituto de Salud Carlos III, 28029 Madrid, Spain; 4 Vascular Surgery Department, Vall d’Hebron Hospital Campus, 08035 Barcelona, Spain; nicolas.allegue@vallhebron.cat (N.A.); anna.gene@vallhebron.cat (A.G.); 5 Orthopedics and Traumatology Department, Vall d’Hebron Hospital Campus, 08035 Barcelona, Spain; andrea.sallent@vallhebron.cat; 6 Physical Medicine and Rehabilitation Department, Vall d’Hebron Hospital Campus, 08035 Barcelona, Spain; almudena.crespo@vallhebron.cat (A.C.); danielamaria.issa@vallhebron.cat (D.I.); 7 Reconstructive Surgery Department, Vall d’Hebron Hospital Campus, 08035 Barcelona, Spain; jordi.serracanta@vallhebron.cat (J.S.); daniloantonio.rivas@vallhebron.cat (D.R.); 8 Radiodiagnosis Department, Vall d’Hebron Hospital Campus, 08035 Barcelona, Spain; carme.torrents@vallhebron.cat (C.T.); mariateresa.veintemillas.idi@gencat.cat (M.T.V.); 9 Infectious Diseases Department, Vall d’Hebron Hospital Campus, 08035 Barcelona, Spain; nuria.fernandezhidalgo@vallhebron.cat; 10 CIBER de Enfermedades Infecciosas (CIBERINFEC), Instituto de Salud Carlos III, 28029 Madrid, Spain; 11 Universitat Autònoma de Barcelona, 08193 Barcelona, Spain

**Keywords:** diabetes, diabetic complications, diabetic foot ulcer, lower-extremity amputations

## Abstract

Background: One of the most devastating complications of diabetes is diabetes-related foot disease (DFD), which is a priority for public health systems. The 2016–2020 Catalonia Health Plan aimed to reduce the incidence of total and major lower-extremity amputations (LEAs) due to DFD by 10% in the population aged 45–74 years. The aim of the present study was to compare the incidence of LEA-DFD 5 years before and after the creation of the Diabetic Foot Multidisciplinary Unit at our Hospital. Methods: We prospectively collected all cases of LEA-DFD performed at Vall d’Hebron University Hospital from 1 January 2016 to 31 December 2020. Cases of LEA-DFD performed from 1 January 2011 to 31 December 2015 were retrospectively reviewed. The incidence of LEA-DFD between these periods was compared. Results: A total of 457 LEAs due to DFD were performed in 316 patients. We observed a reduction of 27.9% [CI: 23.7–32.1%] in the incidence of total LEA in the 2016–2020 period in comparison with the period 2011–2016 (0.8 ± 0.1 vs. 1.1 ± 0.3 per 10.000 inhabitants/year, *p* < 0.001), as well as a reduction of 49.3% [CI: 44.6–53.9%] in the incidence of major LEA-DFD (0.15 ± 0.1 vs. 0.30 ± 0.1 per 10.000 inhabitants/year, *p* < 0.001). Conclusions: The implementation of a Diabetic Foot Multidisciplinary Unit resulted in a significant reduction in the rate of amputations due to DFD in the population with diabetes in North Barcelona.

## 1. Introduction

Diabetes, one of most prevalent diseases around the world, is a serious, long-term condition with a major impact on the lives and well-being of individuals, families, and societies worldwide. The global diabetes prevalence among people aged 20–79 years in 2021 was estimated to be 10.5% (536.6 million people), rising to 12.2% (783.2 million) in 2045 [1]. One of the most devastating complications of diabetes is diabetic foot. People with diabetes have a 15- to 46-fold higher risk of suffering a lower-extremity amputation (LEA). Diabetic foot amputation represents almost one-quarter of all hospital admission days of people with diabetes, and is the leading cause of non-traumatic amputation in high-income countries [2].

Diabetic foot ulcer (DFU) is the main clinical event prior to amputation in people with diabetes. Approximately 80% of amputations were preceded by a DFU [3], which reduces quality of life due to the presence of pain, impaired mobility, or limiting social activities [4]. Once an LEA has occurred, the risk of a subsequent amputation increases to 25% and 68% after 3 to 5 years of follow-up, respectively [2]. Both DFU and LEA are associated with a significant increase in healthcare cost. For example, in the United Kingdom, direct cost ranged from GBP 3456 for a DFU to GBP 9477 for a LEA, per patient/year (5). In Spain, the reported direct cost was around the EUR 21.875 per LEA [5].

The risk of death at 10 years for a patient with diabetes who has had a DFU is 47% higher than the risk for a patient without a DFU [6]. Five-year mortality rates after a new-onset DFU have been reported between 43% and 55% and up to 74% for patients with an LEA. These rates are higher than those reported for several types of cancer including prostate, breast, colon, and Hodgkin’s disease [7].

DFU requires early diagnosis, careful evaluation, and personalized treatment. Primary care centers (PCC) play a fundamental role in identifying feet at risk of developing LEAs and in the initial treatment of DFUs. A correct evaluation of the patient and transfer to the appropriate tertiary level of care can not only save the leg but also the patient’s life [8]. For these reasons, the care and management of diabetic foot must be a priority for public health systems, and has received more attention in the past decades, culminating in the creation of multidisciplinary outpatient clinics [9].

One of the main objectives of the 2016–2020 Catalonia Health Plan was to reduce total and major LEAs due to diabetes-related foot disease (LEA-DFD) by 10% (expressed as a rate per 10.000 inhabitants) in populations aged 45–74 years [10]. This objective was not accomplished in the previous Catalonian Health Plan (2010–2015). At Vall d’Hebron University Hospital, a Diabetic Foot Multidisciplinary Unit was constituted in November 2015, following the recommendations of the International Diabetic Foot Consensus [11]. One of their purposes was to create protocols for referral from the PCC in our engagement area (Muntanya Primary Care Service, North Barcelona), with the final aim to reduce the rate of LEA-DFD.

The aim of the present study was to evaluate the impact of the creation of a Diabetic Foot Multidisciplinary Unit at our Hospital, by comparing the incidence of LEA-DFD 5 years before (period 2011–2015) and 5 years onwards (period 2016–2020).

## 2. Materials and Methods

### 2.1. Subjects and Methods

We prospectively registered all consecutive cases of LEA-DFD performed at Vall d’Hebron University Hospital from 1 January 2016 to 31 December 2020. Cases of LEA-DFD performed from 1 January 2011 to 31 December 2015 were retrospectively collected. This study was conducted following the Strengthening of the Reporting of Observational Studies in Epidemiology guidelines.

We included adult (≥18 year old) patients with LEA-DFD from the engagement area of Vall d’Hebron University Hospital (Muntanya Primary Care Service, North Barcelona, Spain) which comprises a population of almost 400,000 inhabitants. All patients with type 2 diabetes included in the present study were followed by a GP belonging to the engagement area of Vall d’Hebron University Hospital. In addition, a significant proportion of patients was followed by both the GP at primary care and by an endocrinologist at Vall d’Hebron University Hospital (36.1% of patients from the period 2011–2015 and 41.4% of patients from the period 2016–2020). Almost all patients with type 1 diabetes were followed by an endocrinologist at Vall d’Hebron University Hospital (78% of patients from the period 2011–2015 and 100% of patients from the period 2016–2020).

A minor amputation was defined as any LEA distal to the ankle joint (ICD-9-CM codes 84.10–84.12), and a major amputation was defined as any LEA through or proximal to the ankle joint (ICD-9-CM codes 84.13–84.17).

All clinical information was collected using the digitalized clinical history. We collected data from the participant’s medical history records related to the following variables: socio-demographic characteristics, toxic habits (smoking), cardiovascular risk factors (hypertension, hyperlipidemia, obesity, previous history of stroke and ischemic heart disease), data on diabetes (duration, HbA1c, diagnosis of diabetic microvascular complication such as retinopathy, chronic kidney disease, and peripheral neuropathy), and previous history of amputation or DFU. DFU was defined as a full-thickness lesion below the ankle, regardless of whether the patient presented with neuropathy and/or peripheral artery disease [3].

We calculated the yearly age-specific incidence rates (per 10.000 inhabitants) by dividing the number of cases per year by the corresponding number of people in that population territory, according to data from the National Institute of Statistics and census (https://ajuntament.barcelona.cat/estadistica/castella/Estadistiques_per_temes/Poblacio_i_demografia/Poblacio/Xifres_oficials_poblacio/a2017/edat/index.htm, accessed on 12 October 2021). For calculating the incidence in patients with diabetes, we estimated a prevalence of diabetes of 14% in our reference area following the data published in the di@betes.es study [12].

This study was conducted in agreement with the Declaration of Helsinki ethical principles for medical research involving human subjects.

### 2.2. Statistical Analysis

Categorical variables were expressed as percentage. Quantitative variables that follow a normal distribution were expressed as means and standard deviation; for those that did not, they were expressed as median and range. The chi-square test was used for comparisons between qualitative variables. For the quantitative variables that follow a normal distribution, the Student *t*-test was used. The non-parametric Kruskal–Wallis test was used to evaluate the differences between groups of variables that do not follow a normal distribution. Significance was set at *p* < 0.05. Statistical analyses were performed with the STATA 15 statistical package.

## 3. Results

A total of 457 LEAs for DFD were performed at Vall d’Hebron University Hospital from 1 January 2011 to 31 December 2020. These LEA-DFD cases were performed in 316 individuals (141 were re-amputations). Eight (1.8%) were in people <45 years, 302 (66.1%) in people between 45–75 years, and 147 (32.2%) in people > 75 years.

The mean rate of LEA-DFD over 10 years of analysis was 0.94 per 10.000 inhabitants/year. In the group of people with diabetes between 45–75 years, mean rate of LEA-DFD were 18.4 per 10.000 inhabitants/year.

The main characteristics of patients at the time of LEA-DFD according the period analyzed are shown in Table 1. Subjects from the prospective cohort 2016–2020 had a significant lower prevalence of hypertension and microvascular diabetic complications in comparison with the 2011–2015 cohort.

The main characteristics of the LEA-DFD cases are detailed in Table 2. In the 2016–2020 cohort, the proportion of major amputations decreased, but the percentage of minor amputations increased. The percentage of revascularization procedures performed before LEA-DFD was significantly higher in the 2016–2020 cohort in comparison with the 2011–2015 cohort.

**Figure 1:**
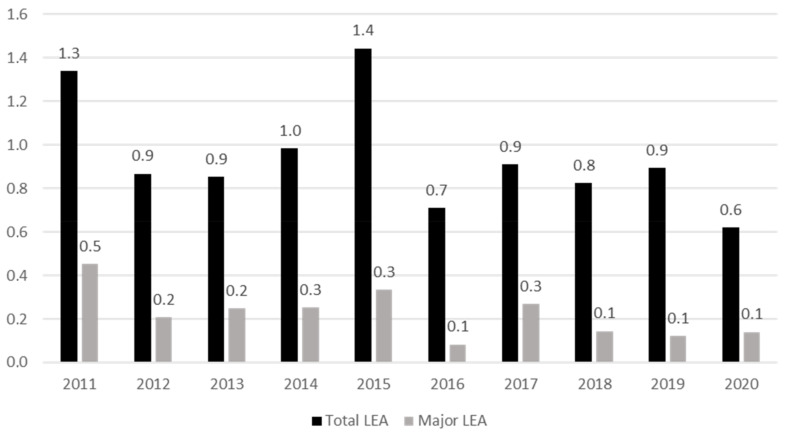
Incidence of total and major LEA-DFD cases per 10.000 inhabitants/year.

The incidence of total and major LEA-DFD per 10.000 inhabitants/year is shown in Figure 1. We observed a reduction of 27.9% [CI: 23.7–32.1%] in mean rate of total LEA in the 2016–2020 period in comparison with the 2011–2016 period (1.1 ± 0.3 vs. 0.8 ± 0.1 per 10.000 inhabitants/year, *p* < 0.001). Furthermore, we observed a reduction of 49.3% [CI: 44.6–53.9%] in mean rate of major amputations in the 2016–2020 period in comparison with the 2011–2016 period (0.30 ± 0.1 vs. 0.15 ± 0.1 per 10.000 inhabitants/year, *p* <0.001).

**Figure 2:**
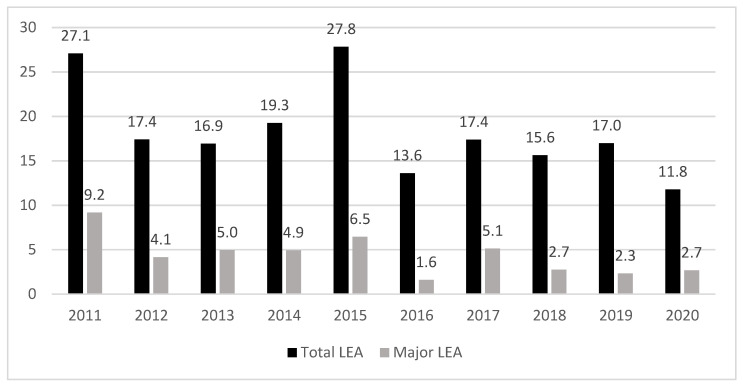
Incidence of total and major LEA-DFD cases between 45–75 year per 10.000 inhabitants with diabetes/year.

The incidence of total and major LEA-DFD cases between 45–75 years per 10.000 subjects with diabetes/year is displayed in Figure 2. We found a reduction of 30.5% [CI: 25.3–35.9%] in the mean rate of total LEA-DFD cases in people with diabetes between 45–75 year in the 2016–2020 period in comparison with the 2011–2016 period (21.7 ± 5.3 vs. 15.1 ± 2.4 per 10.000 inhabitants/year, *p* <0.001). In addition, we observed a reduction of 51.3% [CI: 44.5–57.1%] in the mean rate of major LEA-DFD cases in the 2016–2020 period in comparison with the 2011–2016 period (5.9 ± 2.0 vs. 2.9 ± 1.3 vs. per 10.000 inhabitants/year, *p* < 0.001).

## 4. Discussion

In this study, we reported a significant reduction of LEA-DFD incidence after the constitution of the Diabetic Foot Multidisciplinary Unit, not only in total of number of amputations (27.9%), but also in major amputations (49.3%). This reduction was even higher when the incidence of LEA-DFD was evaluated in people with diabetes aged 45–75 years (reductions of 30.5% for total amputations and 51.3% for major amputations). With these results, we achieved the objective of the Health Plan of Catalonia 2015–2020 regarding diabetic foot.

In 2014, the Spanish Diabetic Foot Group of the Spanish Diabetes Society [15] analyzed the reasons for the poor results on reducing LEA-DFD incidence. The main explanations proposed were the shortage of diabetic foot units in Spain and the limitation for access to podiatrists in the National Health System, because they are not included among the professionals hired by the Spanish public health system. Only one out of four patients with diabetes in Spain could benefit from care in a multidisciplinary diabetic foot unit [16] following the recommendations of the International Diabetic Foot Consensus [17].

In Spain, the incidence of minor and major amputations decreased significantly from 2001 to 2008 (from 0.88 to 0.43 per 100,000 inhabitants and from 0.59 to 0.22 per 100,000 inhabitants, respectively) in patients with type 1 diabetes [18]. However, in patients with type 2 diabetes, the incidence of minor and major LEA-DFD increased significantly (from 9.23 to 10.9 per 100,000 inhabitants and from 7.12 to 7.47 per 100,000 inhabitants, respectively) [18]. More recent reports by Rubio et al. have shown a significant reduction in the rate of major LEA-DFD in the diabetic population (33%) from the area of Madrid after setting up a Diabetic Foot Multidisciplinary Unit [16,19]. The same group reported an increase in the incidence of LEA-DFD in two consecutive periods (1997–2000 and 2001–2006) previous to the implementation of the Diabetic Foot Multidisciplinary Unit [20,21,22]. In Murcia, another area in Spain, a significant reduction in total and major amputations rates occurred over a 15-year period following improvements in foot care services including an integrated care pathway and a diabetic foot clinic [23].

In the U.S., after years of decline, the age-adjusted rate of non-traumatic LEA per 1000 adults with diabetes increased by 50% between 2009 and 2015, particularly in young and middle-aged adults (3.07 [95% CI 2.79–3.34] vs. 4.62 [95% CI 4.25–5.00], respectively) [24]. Another study found that the increase in the incidence of diabetes-related non-traumatic LEAs from 2000 to 2017 also occurred in older populations, but with a less severe rate than among younger adults (<65 years) [25]. In Australia, analysis of data from the Western Australian Data Linkage System for the period from 2000 to 2010 revealed decreases in the rate of major and minor amputations of 6.2% and 0.6% per year, respectively [26]. In England, data from the National Health Service comprising all patients with non-traumatic amputations in the period from 2004 to 2008 showed that the incidence of LEA-DFD did not significantly change (from 27.5 to 25.0 per 10.000 subjects with diabetes) [27]. During the same period, in Ireland, total diabetes-related amputation rates increased non-significantly during the study period (144.2 in 2005 to 175.7 in 2009 per 100,000 people with diabetes) [28]. In Italy, from 2001 to 2010, a progressive reduction in amputation rates was observed for major amputations (30.7%), while the rates of minor amputations were nearly stable [29]. In Germany [30], an estimated reduction in amputations above the toe level by 37.1% over 15 years (1990–2005) has been reported. In Finland, the standardized population-corrected rate of first major LEA per 100,000 person-years declined significantly from 10.0 to 7.3, while the minor-major LEA ratio progressed from 0.86 to 1.35 [31].

The significant reduction in LEA-DFD that we observed since the implementation of the Diabetic Foot Multidisciplinary Unit could be substantially attributed to the increase in the number of revascularization procedures, the incorporation of a podiatrist at a diabetes unit, the development of a consensual protocol for referral to the diabetic foot unit from primary care centers, and the higher coordination among the professionals involved in the diagnosis and treatment of diabetic foot. The relative short time period in which the comparison was performed (two consecutive period of 5 years each) makes the significant influence of other factors unlikely, apart from the Diabetic Foot Multidisciplinary Unit impact. Nevertheless, the improvement of microangiopathic complications alongside the study period might also play a specific role that should be considered.

The limitations of the present work are the retrospective nature of part of the study and the unicentric inclusion of patients. The lack of data analysis comparing data with other tertiary hospitals without Diabetic Foot Multidisciplinary Unit precludes the ability to generalize the obtained results.

In conclusion, the task performed by a Diabetic Foot Multidisciplinary Unit resulted in a clinically and statistically significant reduction in the rate of LEA-DFD in North Barcelona (Muntanya Primary Care Service). We should redouble efforts in the foot care of people with diabetes who are at risk of developing a DFU because reduction in the risk of foot ulceration also reduces the risk of infection, hospitalization, and lower-extremity amputation. The role of primary PCC in identifying the foot at risk and the timely referral to the diabetic multidisciplinary foot will be crucial for the success of programs aimed at reducing LEA-DFD.

## Figures and Tables

**Table 1:** Patients’ characteristics according the period analyzed.

	Period 2011–2015 (n = 147)	Period 2016–2020 (n = 169)	*p*
Number of total amputations	264	193	
Number of amputations/patients	1.79	1.14	<0.001
Age (years), mean ± SD	66.9 (0.9)	68.5 (0.9)	0.234
Sex (men), n (%)	103 (70.1)	125 (73.9)	0.132
Body mass index (BMI) (kg/m^2^), mean ± SD	28.6 ± 0.4	29.1 ± 0.4	0.408
Smoker, n (%)	44 (29.9)	46 (27.2)	0.111
Former smoker, n (%)	47 (31.9)	50 (29.6)	0.608
Cigarettes packs (20 cigarettes/day), mean ±SD	1.07 ± 0.8	1.1 ± 0.1	0.690
Hypertension (HTA), n (%)	139 (94.6)	151 (89.4)	0.022
Dyslipidemia (DLP), n (%)	126 (85.7)	136 (80.5)	0.116
Ischemic heart disease, n (%)	56 (38.1)	62 (36.7)	0.680
Stroke, n (%)	24 (16.3)	32 (18.9)	0.587
Type 1 Diabetes (%)/Type 2 Diabetes (%)	6.1/93.9	3.1/96.9	0.237
Diabetes duration (years), mean ± SD	18.4 ± 0.8	19.1 ± 0.8	0.505
Insulin user, n (%)	117 (79.6)	118 (69.8)	0.140
Insulin dose (UI/kg/day), mean ± SD	0.54 ± 03	0.51 ± 0.3	0.103
HbA1c (%), mean ± SD	8.2 ± 0.2	8.0 ± 0.1	0.289
HbA1c (mmol/mol), mean ± SD	66.1 ± 21.3	63.9 ± 22.4	0.289
Retinopathy, n (%)	104 (70.8)	104 (61.5)	0.015
VTDR, n (%)	66 (44.9)	54 (31.9)	0.021
Nephropathy, n (%)	107 (72.8)	100 (59.2)	0.012
Severe nephropathy, n (%)	55 (37.4)	14 (8.3)	<0.001
Polyneuropathy, n (%)	134 (91.1)	129 (76.3)	0.047

Data are expressed as the mean ± SD or percentage. Vision-Threatening Diabetic Retinopathy (VTDR) includes severe non-proliferative diabetic retinopathy (NPDR), proliferative DR, and diabetic macular edema (DME) [13]. Severe nephropathy was defined as severe chronic kidney disease (CKD) (GFR < 30 mL/min/1.73 m^2^), and End-stage kidney disease (ESKD) (dialysis dependency) [14].

**Table 2:** Lower extremity amputation characteristics.

	Period 2011–2015	Period 2016–2020	*p*
Amputations, n	264	193	
Number of major amputations (%)	27.2	19.2	0.047
Number of minor amputations (%)	72.7	80.8	0.047
Previous amputations (%)	80.8	60.9	<0.001
Previous lower-limb revascularization (%)	51.1	65.8	0.021
Previous diabetic foot ulcer (%)	87.5	84.9	0.124
Charcot foot (%)	4.6	3.6	0.173

Data are expressed as number or percentage.

## Data Availability

Data are available from the corresponding authors upon request.
